# Influences of Exposure Time and Mortality Assessment Interval on Bioassay Results of Insecticide-Resistant Tropical Bed Bugs (Hemiptera: Cimicidae)

**DOI:** 10.3390/insects11090640

**Published:** 2020-09-18

**Authors:** Xin-Yeng Leong, G. Veera Singham, Alexander Chong Shu-Chien, Stephen L. Doggett, Chow-Yang Lee

**Affiliations:** 1 Urban Entomology Laboratory, Vector Control Research Unit, School of Biological Sciences, Universiti Sains Malaysia, Minden 11800, Penang, Malaysia; leongxinyeng@gmail.com; 2 Centre for Chemical Biology, Universiti Sains Malaysia, Bayan Lepas 11900, Penang, Malaysia; veerasingham@usm.my (G.V.S.); alex@usm.my (A.C.S.-C.); 3 School of Biological Sciences, Universiti Sains Malaysia, Minden 11800, Penang, Malaysia; 4 Department of Medical Entomology, NSW Health Pathology-ICPMR, Westmead Hospital, Westmead, NSW 2145, Australia; Stephen.Doggett@health.nsw.gov.au; 5 Department of Entomology, University of California, 900 University Avenue, Riverside, CA 92521, USA

**Keywords:** tropical bed bug, insecticide resistance, exposure time, mortality assessment interval

## Abstract

**Simple Summary:**

Bed bugs cause health and economic impacts due to their hematophagous behavior. The tropical bed bug, *Cimex hemipterus* (F.) is predominant in tropical and subtropical regions and modern strains demonstrate high levels of insecticide resistance making them a major challenge to control. In this present study, we investigated the effect of exposure time and mortality assessment interval on bioassay results. Bed bugs were treated separately with six insecticide products at label rates using the surface contact method, with multiple exposure times (5 min, 10 min, 30 min, 1 h, 4 h, and continuous exposure for 96 h or 14 days for Phantom). Post-treatment mortalities of bed bugs were assessed daily for up to 4 days (Tandem, Temprid SC, Pesguard FG161, Sumithrin, and Sumithion) and 14 days for Phantom. Results showed that longer exposure times led to rapid knockdown and better killing effect in bed bugs. Exposure times and mortality assessment intervals should be carefully considered in resistance and efficacy studies with insecticide products.

**Abstract:**

We evaluated the influences of insecticide exposure time and mortality assessment intervals on the bioassay results of three insecticide-resistant tropical bed bug *Cimex hemipterus* (F.) populations (Madam Mo, Tanjong Tokong, and Green Lane). This was achieved using the surface contact method and tested with six commercial insecticide products: Tandem, Temprid SC, Pesguard FG161, Sumithrin, Sumithion, and Phantom applied at label rate on glass petri dishes. Six exposure times (5 min, 10 min, 30 min, 1 h, 4 h, and continuous exposure for 4 or 14 days for Phantom) were tested. A susceptible common bed bug *Cimex lectularius* L. strain (Monheim) was used as the reference strain as no susceptible *C. hemipterus* strain exists. In treatment with Temprid SC, the Tanjong Tokong strain showed significantly higher KT_50_ values at 5, 10, and 30 min exposures than 1 h, 4 h, and continuous exposures. When all resistant populations were evaluated at continuous exposure to Sumithion, they demonstrated moderate resistance levels (RR_50_ ranged from 6.0 to 7.9), while KT_50_ and KT_95_ of other shorter exposure times failed to be generated due to low knockdown rate. Higher mortalities were observed in Tanjong Tokong and Green Lane strains when tested at longer exposure times with Temprid SC, Pesguard FG161, Sumithrin, Sumithion, and Phantom. Better killing effect was observed in the treatment with Temprid SC (Tanjong Tokong and Green Lane strains), Pesguard FG161 (Tanjong Tokong and Green Lane strains), Sumithrin (all *C. hemipterus* strains), Sumithion (all *C. hemipterus* strains), and Phantom (all strains tested) at longer mortality assessment intervals. We demonstrated that insecticide exposure time and mortality assessment interval could potentially affect outcomes of product performance evaluations, resulting in underestimation or overestimation of insecticide resistance levels in field populations.

## 1. Introduction

The global resurgence of bed bugs has been a growing concern over the last two decades. The two species of bed bugs, namely the common bed bug (*Cimex lectularius*) and the tropical bed bug (*Cimex hemipterus*), are frequently associated with humans [1,2]. Bed bugs and their bites can cause a range of medical issues in humans, such as skin irritations, systemic reactions, and occasionally iron deficiency when the insect is numerous [2,3]. The monitoring and eradication of bed bug infestations is difficult due to their cryptic behavior and high levels of insecticide resistance. In spite of the resistance, insecticides continue to play a crucial role in bed bug management.

Insecticide resistance in bed bugs has been evaluated using technical grade insecticides [4,5,6,7] and commercial insecticide products and formulations [8,9,10,11]. Bed bugs have been found resistant to several insecticide classes that are commonly incorporated in formulated products, such as pyrethroids [11,12,13], neonicotinoids [4,14], organophosphates [15,16,17], and pyrroles [10]. Several bioassay methods have been used in resistance testing. For instance, dose-response assays with topical application [4,6,7,18], concentration-response assays with a surface contact method [10,19,20,21], and time-response assays with a surface contact method [8,11,17,22], or direct spraying [17]. For laboratory evaluations of commercial insecticide products and formulations, a time-response assay [8,11,23] and endpoint assay [9,24] have been commonly used. Application methods of formulated insecticides, such as direct spraying of diluted products on insects [9,17] and exposing insects on insecticide-treated surfaces [11,24], have been commonly employed.

There have been limited studies on the effect of exposure time on treated surface and mortality assessment intervals on time response assays tested with a single insecticide application rate. Previous reports on insecticide formulations were evaluated at fixed exposure times on treated surfaces, ranging from the shortest being 5 min exposure [24] to continuous contact on treated surface until the experiment ended [8,22]. Several studies comparing exposure times have been undertaken on *C. lectularius* [24,25], *Anopheles* spp. [26], as well as *Culicoides* spp. [27], and differences were observed in insecticides uptake, knockdown responses, and mortalities.

Various published sources or guidelines on insecticide resistance monitoring suggests different mortality assessment intervals for time response assays with a single insecticide application rate. WHO [28] suggests 24 h for malaria vectors with fast-acting insecticides and beyond 24 h for slow-acting insecticides. For bed bug evaluations, Lee and colleagues [29] stated that 24 or 48 h could be used as an endpoint to assess mortality for fast-acting insecticides (pyrethroids and organophosphates) and 14 days for slow acting insecticides, such as, chlorfenapyr. The US EPA guidelines [30] recommends 2, 24, 48, 72, and 96 h for bed bug pesticide product performance evaluations. Singh et al. (2016b) [9], Wang et al. (2016b) [24], and Ashbrook et al. (2017) [10] observed mortalities of *C. lectularius* up to 2 weeks post-treatment with the slow-acting insecticides, chlorfenapyr.

The control of bed bugs relies heavily on residual insecticides. Nonetheless, the contact period of bed bugs on the treated surface is unknown, due to their nocturnal and cryptic behavior in nature. Insecticide resistance evaluation with different exposure times and mortality assessment intervals may need to be tested to better reflect insecticide efficacies in field condition. Similar studies have been conducted by Vander Pan et al. (2019) [21] on different assessment intervals of insecticide products using field strains of *C. lectularius*. Wang et al. (2016b) also conducted similar experiments with different exposure times and assessment intervals of insecticide products against field *C. lectularius* [24]. Nonetheless, no similar study has been reported on *C. hemipterus*.

This study evaluated the influence of exposure time (5 min, 10 min, 30 min, 1 h, 4 h, and continuous exposure) and mortality assessment interval on the bioassay results of different insecticide products when applied at the label rate. The evaluations were carried out using a surface contact method on adult field strains of *C. hemipterus* and a laboratory susceptible strain of *C. lectularius*. Knockdown responses and mortality were recorded for up to 4 days with the fast-acting insecticide products (Tandem, Temprid SC, Pesguard FG161, Sumithrin, and Sumithion) and up to 14 days for the slow-acting product (Phantom).

## 2. Materials and Methods

### 2.1. Insects

Three *C. hemipterus* field strains (Madam Mo, Tanjong Tokong, and Green Lane) collected in Penang, Malaysia were evaluated in this study (Table 1). A susceptible *C. lectularius* strain, Monheim, was chosen as the reference strain as no susceptible *C. hemipterus* strain could be sourced worldwide. All bed bug strains were reared in 0.5-L glass containers with folded craft papers as harborages, under conditions of 27 ± 2 °C, 70% ± 5% RH and a photoperiod of 12:12 (L: D). Bed bugs were fed defibrinated rabbit blood (No. of animal ethics approval: USM/Animal Ethics Approval/2016/(104) (819)) once per week using the Hemotek membrane feeding system (Discovery Workshops, Accrington, UK). Bed bugs were fed 7–9 days prior to experiment.

### 2.2. Chemical Preparations

Six insecticide products were tested in this study (Table 2). The products were diluted to label rate using deionized water. The diluted insecticide was applied onto a glass Petri dish (diameter: 90 mm) based on the application rate (Table 2) and spread evenly. Control Petri dishes were treated with deionized water only. The treated Petri dishes were placed in a fume hood and allowed to dry overnight. The wall of the treated Petri dishes was coated with a thin layer of Fluon (polytetrafluoroethylene suspension; BioQuip, Rancho Dominguez, CA) to prevent the insects from escaping.

### 2.3. Surface Contact Assays

Six experiment sets with different exposure times were used and included 5 min, 10 min, 30 min, 1 h, 4 h, and continuous exposure. For Tandem, Temprid SC, Pesguard FG161, Sumithrin and Sumithion, continuous exposure was up to 4 days, while for Phantom, it was 14 days. Ten adult bed bugs were introduced into each treated Petri dish (sex ratio 1:1). After the designated exposure time, the treated bed bugs were removed (except for continuous exposure) and placed into clean Petri dishes with folded filter paper as a harborage. Knockdown responses of the treated bed bugs were observed at regular time intervals (5 min interval for the first hour, 30 min interval for first 6 h and subsequently 6 h interval until experiment ended) for up to 4 days (for Tandem, Temprid SC, Pesguard FG161, Sumithrin, and Sumithion) and up to 14 days (for Phantom). A bed bug was considered knocked down when it could not right itself up after being gently probed with a pair of soft forceps. The knocked down bed bugs were kept in a clean container during the first 24 h in case any insects recovered. Mortalities of the knocked down insects (defined as death rate of tested insects) were scored after the 24 h period, and subsequently every day until the experiment ended. The knocked down bed bugs were considered dead when they showed no movement or were in a moribund state (on their back and with uncoordinated movement) after being gently probed with a pair of soft forceps after 24 h. Each experiment set was replicated three times.

### 2.4. Statistical Analysis

Control knockdown and mortality were corrected using Abbott formula [31] and subjected to probit analysis using Polo Plus [32]. Knockdown time (KT_50_ and KT_95_) for bed bugs were generated using time-response data. The resistance ratio (RR_50_) was calculated by dividing KT_50_ values of resistant strain with that of the corresponding value of the Monheim strain. χ^2^ goodness-of-fit tests were used to confirm whether the data set conformed with the assumptions of probit model [5]. KT_50_ and KT_95_ values were considered significantly different when their 95% fiducial limits (FLs) did not overlap [33,34]. The resistance level classification followed that of Leong et al. [11] and Lee and Lee [35] (Table 3). As the data did not meet the assumptions of normality and homogeneity of variance after arcsine and square root transformation, they were subjected to non-parametric tests. Kruskal–Wallis test (*p* = 0.05) and pairwise multiple comparisons Dunn’s test (*p* = 0.05) were used to analyze the effect of exposure time towards percentage mortalities of bed bugs at 1 and 4 days post-treatment for the fast-acting products (Tandem, Temprid SC, Pesguard FG161, Sumithrin and Sumithion), at 1, 7, and 14 days post-treatment for the slow-acting product (Phantom). Friedman test (*p* = 0.05) and pairwise multiple comparisons Dunn’s test (*p* = 0.05) were used to analyze the effect of mortality assessment interval towards percentage mortalities of bed bugs. All tests were performed using statistical package SPSS v24 (IBM Corp., Armonk, NY).

## 3. Results

### 3.1. Surface Contact Assay

#### 3.1.1. KT_50_ and KT_95_ Values of Bed Bugs Exposed to Different Exposure Times

Monheim and Madam Mo strains showed no significant differences between all KT_50_ and KT_95_ values tested on Tandem at different exposure times. Green Lane strain showed significantly larger KT_50_ values in 5 and 10 min than 1 h, 4 h, and continuous exposure (Table 4). No significant differences between exposure times were observed in Monheim and Madam Mo strains when treated with Temprid SC (Table 4). However, Tanjong Tokong strain showed substantially larger KT_50_ and KT_95_ values when exposed to Temprid SC for 5, 10, and 30 min than other exposure times tested (Table 4). Green Lane strain exhibited substantially lower KT_50_ values in treatment with Temprid SC at 4 h and continuous exposure than that of 5 min, 10 min, 30 min, and 1 h (Table 4).

When exposed to pyrethroid only based products (Pesguard FG161 and Sumithrin), Monheim strain showed no significant differences between all exposure times tested (Table 4). Madam Mo strain demonstrated low resistance levels at all exposure times towards both Pesguard FG161 and Sumithrin, with 5 min exposure to Pesguard FG161 showed significantly larger KT_50_ values compared to other exposure times tested (Table 4). Tanjong Tokong and Green Lane strains were very highly resistant towards Pesguard FG161 and Sumithrin at all exposure times tested. The KT_50_ and KT_95_ values of all exposure times tested with both pyrethroid-based products failed to be generated for both Tanjong Tokong and Green Lane strains, except KT_50_ values continuous exposure for Tanjong Tokong strain (Table 4).

In treatment with Sumithion, the KT_50_ values of 5 min exposure for the Monheim strain was significantly higher than 30 min, 1 h, 4 h, and continuous exposure (Table 4). Monheim strain also showed significantly higher KT_95_ values at 5 min exposure than 1 h, 4 h, and continuous exposure to Sumithion. KT_50_ and KT_95_ values of Sumithion for all resistant strains failed to be generated, due to limited knockdown responses (except for continuous exposure) (Table 4). However, all resistant strains showed moderate resistance level to Sumithion at continuous exposure (Table 4).

Monheim strain demonstrated significantly lower KT_50_ values at 30 min, 1 h, 4 h, and continuous exposure to Phantom than 5 and 10 min exposure, but no significant difference was observed in KT_95_ values (Table 5). Madam Mo strain showed substantially lower KT_50_ values at 4 h and continuous exposure than 5 min in treatment with Phantom (Table 5). Tanjong Tokong strain showed significantly lower KT_50_ values when tested at 30 min, 1 h, 4 h, and continuous exposure than 5 and 10 min exposure to Phantom (Table 5). Green Lane strain showed 100% knockdown after 14 days post-treatment for 4 h and continuous exposure, while less than 40% knockdown responses were shown for 5 and 10 min exposure.

#### 3.1.2. The Influence of Exposure Time on Percentage Mortalities of Bed Bugs at 1 and 4 Days Post-Treatment

**Figure 1:**
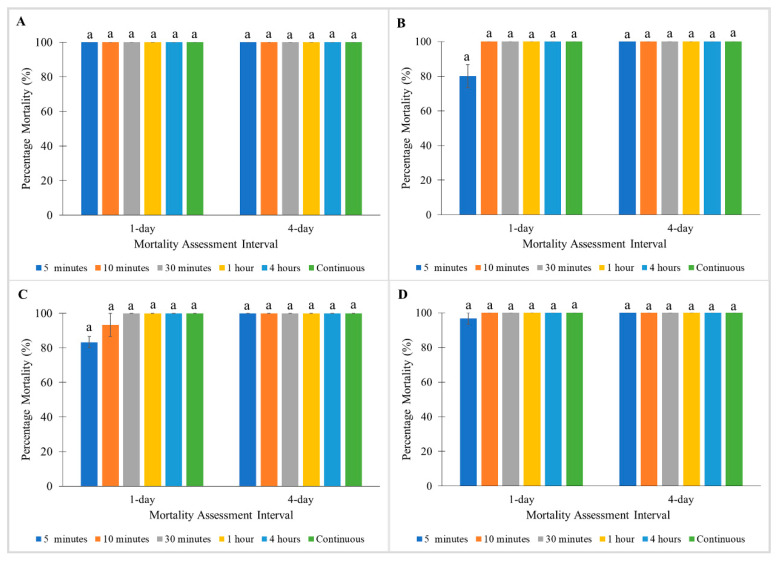
Influence of exposure time on Tandem on percentage mortalities of a susceptible *C. lectularius* strain and three *C. hemipterus* strains at 1 and 4 days post-treatment. (**A**) Monheim, (**B**) Madam Mo, (**C**) Tanjong Tokong, (**D**) Green Lane strains. Bars with different letters are significantly different (Kruskal–Wallis test, *p* < 0.05).

**Figure 2:**
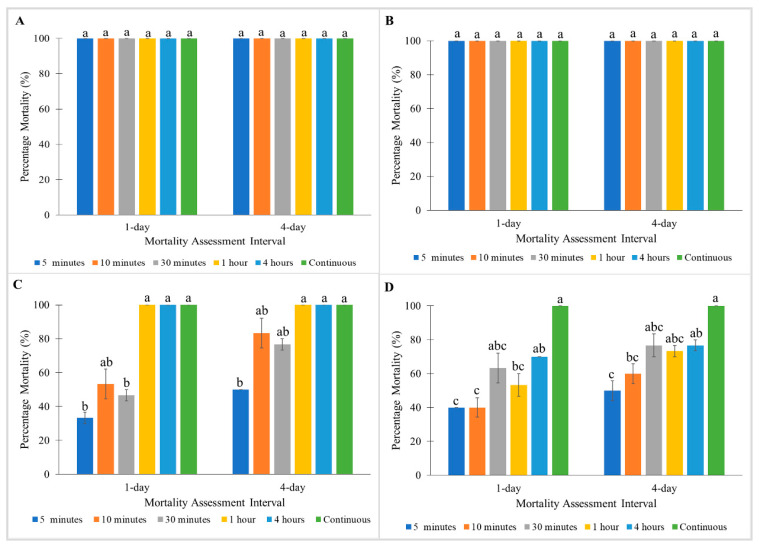
Influence of exposure time on Temprid SC on percentage mortalities of a susceptible *C. lectularius* strain and three *C. hemipterus* strains at 1 and 4 days post-treatment. (**A**) Monheim, (**B**) Madam Mo, (**C**) Tanjong Tokong, (**D**) Green Lane strains. Bars with different letters are significantly different (Kruskal–Wallis test, *p* < 0.05).

All strains exhibited no significant differences between mortalities tested at different exposure times with Tandem (Figure 1). Similarly, no significant differences between mortalities were observed in the Monheim and Madam Mo strains when treated with Temprid SC (Figure 2A,B). Nonetheless, significant differences were found between mortalities tested at different exposure times for Tanjong Tokong (Kruskal–Wallis test: 1 day: χ^2^ = 16.076; df = 5; *p* < 0.05, 4 day: χ^2^ = 14.976; df = 5; *p* < 0.05) and Green Lane strains (Kruskal–Wallis test: 1 day: χ^2^ = 14.3; df = 5; *p* < 0.05, 4 day: χ^2^ = 14.056; df = 5; *p* < 0.05) in treatment with Temprid SC (Figure 2C,D).

**Figure 3:**
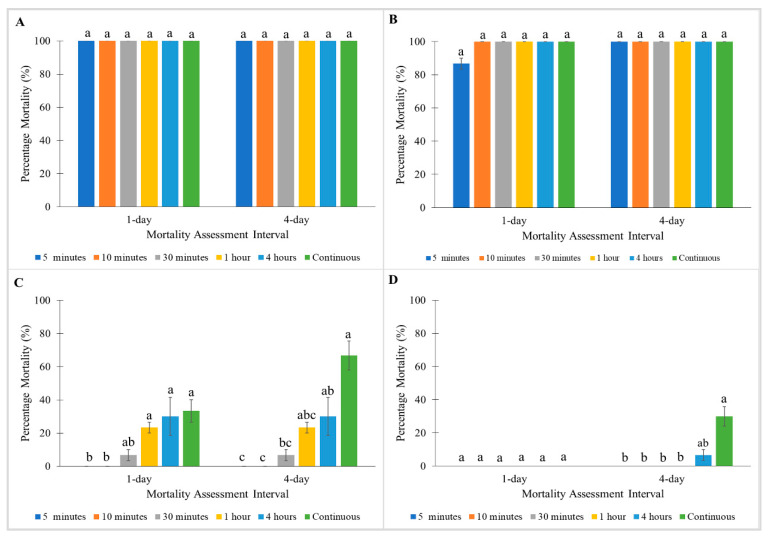
Influence of exposure time on Pesguard FG161 on percentage mortalities of a susceptible *C. lectularius* strain and three *C. hemipterus* strains at 1 and 4 days post-treatment. (**A**) Monheim, (**B**) Madam Mo, (**C**) Tanjong Tokong, (**D**) Green Lane strains. Bars with different letters are significantly different (Kruskal–Wallis test, *p* < 0.05).

**Figure 4:**
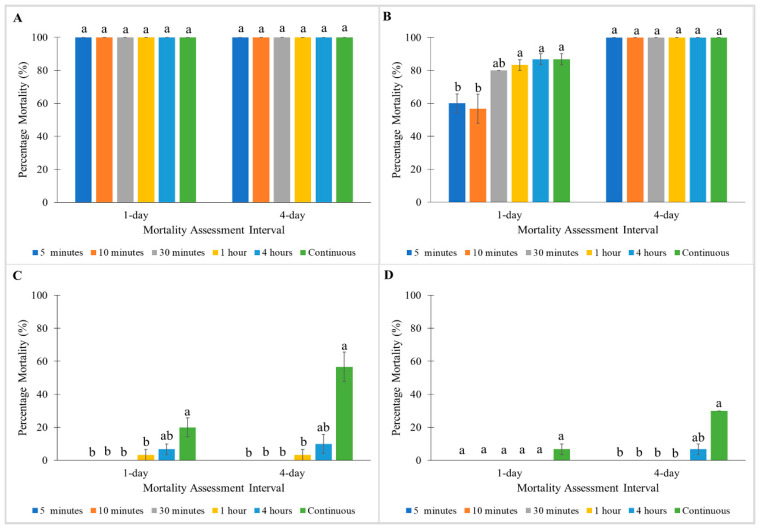
Influence of exposure time on Sumithrin on percentage mortalities of a susceptible *C. lectularius* strain and three *C. hemipterus* strains at 1 and 4 days post-treatment. (**A**) Monheim, (**B**) Madam Mo, (**C**) Tanjong Tokong, (**D**) Green Lane strains. Bars with different letters are significantly different (Kruskal–Wallis test, *p* < 0.05).

For treatment with Pesguard FG161 and Sumithrin, the Monheim strain showed 100% mortalities towards all exposure times at 1 and 4 days post-treatment (Figure 3A and Figure 4A). When tested with Pesguard FG161, Madam Mo strain demonstrated no significant differences between all exposure times at 1 and 4 days post-treatment (Figure 3B). For Sumithrin, exposure times caused a significant effect on 1 day post-treatment mortalities of the Madam Mo strain (Kruskal–Wallis test: 1 day: χ^2^ = 13.629; df = 5; *p* < 0.05) (Figure 4B). Shorter exposure times to Pesguard FG161 and Sumithrin caused no mortality towards pyrethroid-resistant Tanjong Tokong (5 and 10 min exposure) and Green Lane (5 min to 1 h exposure) strains (Figure 3C,D and Figure 4C,D).

**Figure 5:**
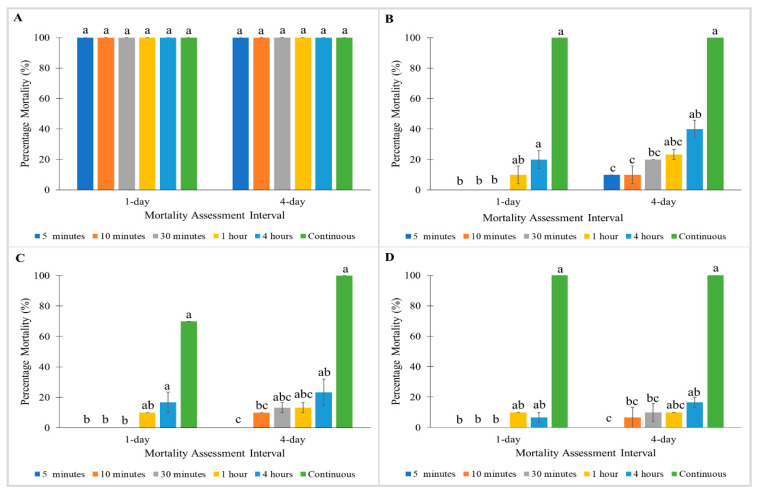
Influence of exposure time on Sumithion on percentage mortalities of a susceptible *C. lectularius* strain and three *C. hemipterus* strains at 1 and 4 days post-treatment. (**A**) Monheim, (**B**) Madam Mo, (**C**) Tanjong Tokong, (**D**) Green Lane strains. Bars with different letters are significantly different (Kruskal–Wallis test, *p* < 0.05).

For Sumithion, no significant differences were observed between mortalities tested at different exposure times in the Monheim strain. Madam Mo (Figure 5B), Tanjong Tokong (Figure 5C), and Green Lane strains (Figure 5D) showed 100% mortalities in continuous exposure at 4 days post-treatment, while shorter exposure times resulted in <40% mortalities.

**Figure 6:**
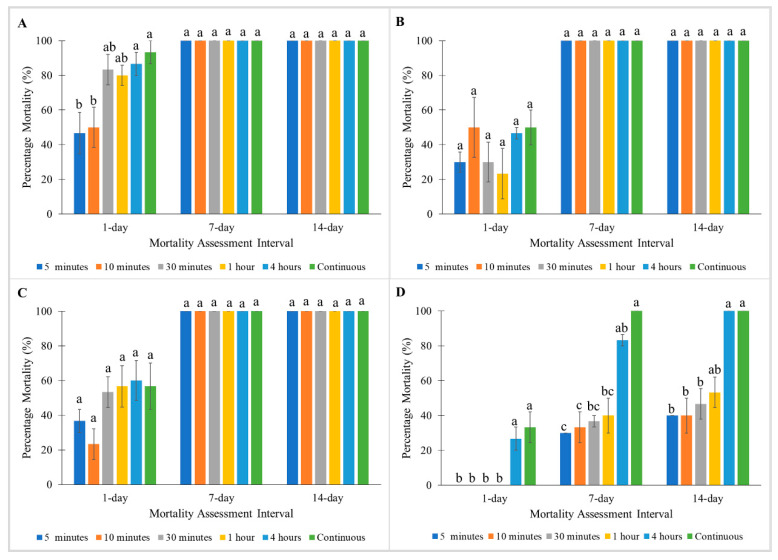
Influence of exposure time on Phantom on percentage mortalities of a susceptible *C. lectularius* strain and three *C. hemipterus* strains at 1, 7, and 14 days post-treatment. (**A**) Monheim, (**B**) Madam Mo, (**C**) Tanjong Tokong, (**D**) Green Lane strains. Bars with different letters are significantly different (Kruskal–Wallis test, *p* < 0.05).

When treated with Phantom, exposure times significantly affected 1 day post-treatment mortalities of the Monheim strain (Kruskal–Wallis test: 1 day: χ^2^ = 11.7; df = 5; *p* < 0.05) (Figure 6A). However, 100% mortalities were observed in the Monheim strain for all exposure times at 7 and 14 days post-treatment with Phantom (Figure 6A). Madam Mo and Tanjong Tokong strains showed 100% mortalities at 7 and 14 days post-treatment with Phantom at all exposure times, but less than 60% mortalities were observed at 1 day post-treatment (Figure 6B,C). At 14 days post-treatment with Phantom, the Green Lane strain showed 100% mortalities when tested with 4 h and continuous exposure to Phantom but less than 60% mortalities were exhibited in shorter exposure times (5 min, 10 min, 30 min, and 1 h exposure) (Figure 6D).

#### 3.1.3. The Effect of Mortality Assessment Intervals towards Percentage Mortalities of Bed Bugs

When treated with Tandem, all strains showed no significant differences between mortalities observed at different assessment intervals (Table S1). For Temprid SC, only the Tanjong Tokong strain exhibited significant differences between mortalities assessed at different intervals (Friedman test: 5 min: χ^2^ = 8.538; df = 3; *p* < 0.05; W = 0.949, 10 min: χ^2^ = 8.76; df = 3; *p* < 0.05; W = 0.973, and 30 min: χ^2^ = 8.769; df = 3; *p* < 0.05; W = 0.974), with 1 day post-treatment mortalities significantly lower than 4 days (Dunn’s test, *p* < 0.05) (Table S1). The Monheim strain showed no significant differences between mortalities at different assessment intervals against Pesguard FG161 and Sumithrin (Table S1). The Madam Mo strain demonstrated no significant differences between mortalities assessed at different intervals when exposed to Pesguard FG161 (Table S1). At 5 and 10 min exposure to Sumithrin, the Madam Mo strain showed significantly higher mortalities when assessed at 4 days than 1 day post-treatment (Dunn’s test, *p* < 0.05) (Table S1). The Tanjong Tokong and Green Lane strains showed significantly lower 1 day post-treatment mortalities than 4 days in continuous exposure to Pesguard FG161 and Sumithrin (Dunn’s test, *p* < 0.05) (Table S1). When treated with Sumithion, assessment intervals showed no significant effect towards mortalities for the Monheim and Green Lane strains (Table S1). At 30 min (Friedman test: χ^2^ = 8.333; df = 3; *p* < 0.05; W = 0.926) and 1 h (Friedman test: χ^2^ = 8.76; df = 3; *p* < 0.05; W = 0.973) exposure to Sumithion, the Madam Mo strain exhibited significantly lower 1 day post-treatment mortalities than that of 4 days (Dunn’s test, *p* < 0.05) (Table S1). For Phantom, assessment time intervals showed a significant effect towards mortalities of all strains, except 30 min, 1 h, 4 h, and continuous exposure in the Monheim strain (Table S2).

## 4. Discussion

This study investigated the influence of exposure time and mortality assessment interval on the bioassay results for three field strains of *C. hemipterus* collected in Penang, Malaysia. The Tanjong Tokong and Green Lane strains in this study showed higher knockdown responses at continuous exposure to Temprid SC, Pesguard FG161, Sumithrin, Sumithion, and Phantom. Higher mortalities were also observed in these strains when longer exposure times were tested, compared to those exposed for shorter times. In a study with *Anopheles* mosquitoes tested on Mosquitoes Contamination Device (MCD) bottle bioassay, longer exposure times also resulted in significantly higher knockdown rates and 24 h mortalities [26]. Similarly, De Keyser et al. [27] reported *Culicoides nubeculosus* (M.) showed substantially lower 24 h mortalities when tested with 10 and 20 min than 60 min on 0.001% deltamethrin-treated paper. According to a study on *C. lectularius*, the uptake of permethrin by the insects significantly increased with longer exposure time and distance travelled on a permethrin-treated mattress liner [25]. Longer exposure times may lead to substantially higher knockdown responses and mortality of bed bugs due to higher uptake of insecticides. With exception to Sumithion and Phantom, all products evaluated in this study contained pyrethroids that could potentially cause locomotor hyperactivity leading to increased acquisition of the lethal concentration of the insecticides [36].

It is also important to note that a longer exposure time could potentially kill all heterozygous resistant insects, hence potentially masking the detection of insecticide resistance. When a longer exposure time is used, this will lead to a higher amount of insecticide contact by the test insects. Moderate resistant strains, normally with a higher number of heterozygous resistant individuals, may have most of the test insects killed after the long exposure, hence masking the ability to detect resistance in these strains. On the other hand, if the exposure time is too short (which leads to lower amount of insecticide absorption), this may lead to overestimation of the resistance level [35,37] which may result in inability to generate KT_50_ and KT_95_ values. Hence, it is vital to choose an optimal exposure time for resistance detection to avoid the issue discussed above.

According to our study, the Green Lane strain showed 100% knockdown and mortality when tested with 4 h and continuous exposure to Phantom but KT_50_ values were unable to be generated for 5 and 10 min exposure. We suggest a judicious choice of exposure time when designing an experiment to avoid an underestimation of the resistance level of an insect population.

In the present study, KT_50_ values of *C. hemipterus* strains for Pesguard FG161 (Tanjong Tokong and Green Lane strains), Sumithrin (Tanjong Tokong and Green Lane strains), Sumithion (Madam Mo, Tanjong Tokong and Green Lane strains), and Phantom (Green Lane strain) failed to be generated, due to the low number of insects knocked down in treatments with shorter exposure times. Low knockdown responses could be either due lower uptake of insecticides due to shorter exposure time (especially for products that contained pyrethroids), or that the tested strain is highly resistant. Bagi et al. [38] reported that time response assay may not be the best approach to test highly resistant strains. This is because in time response assay, the insects were subjected to single insecticide concentration. The concentration used may cause no or low mortality in highly resistant population, leading to failure to generate KT values. On the contrary, the concentration-response assay that subjects the insects to a series of concentrations that resulted in >0–<100% mortalities may be a more suitable approach to evaluate resistance status of the highly resistant population.

Besides, the slower knockdown response also could be due to the insecticide mode of action. For example, chlorfenapyr (Phantom) must first be converted into an active metabolite (AC 303268) through oxidative removal of the N-ethoxymethyl group before the metabolite could inhibit mitochondrial ability to produce ATP [29]. This may be the reason why the insecticide takes longer time to affect the test insects.

Different exposure times may also give the appearance of discrepancies in the performance of a product [24]. The Madam Mo strain demonstrated that 5 and 10 min exposure to Sumithrin had significantly lower 1 day post-treatment mortalities than 1 h, 4 h, and continuous exposure, but all exposure times showed 100% mortalities after 4 days. Wang et al. (2016) [24] also found that a field *C. lectularius* strain tested with 5 min exposure to Phantom at single label rate showed substantially lower mortality than both 4 and 24 h exposures after 7 days post-treatment. However, no significant difference was observed after 15 days post-treatment. Longer exposure times increases the efficacy of a product helping to achieve more rapid knockdown and mortality. Shorter exposure times require a longer time to achieve the knockdown and killing effect. Sternberg et al. (2014) [26] mentioned that observation time interval for mortality could affect the outcomes of a bioassay on *Anopheles* mosquitoes. In the present study, better killing effect was observed in Temprid SC (Tanjong Tokong and Green Lane strains), Pesguard FG161 (Tanjong Tokong and Green Lane strains), Sumithrin (all *C. hemipterus* strains), Sumithion (all *C. hemipterus* strains), and Phantom (all strains) when the post-treatment mortality was assessed at a longer time interval. In contrast, Vander Pan et al. (2019) [21] found that mortality assessment interval had no significant effect on *C. lectularius* mortality using alpha cypermethrin- and bendiocarb-based products. Elbanoby (2019) [39] also found no significant differences between post-treatment mortalities of field collected *C. lectularius* strains when observed at 90 min and 24 h for two pyrethroid-based products, but a significant difference was observed in treatment with a diazinon-based product. Several published sources acknowledged that experiment duration is crucial and should be selected based on the nature of the insecticides (fast- or slow-acting), to allow sufficient time for the intoxication to take place [28,29]. Experiment duration of at least up to 96 h for fast-acting insecticides and 14 days for slow-acting insecticides is suggested for future bed bug insecticide resistance assessments and performance evaluations.

It is imperative to determine whether knockdown rate, mortality rate, or both are more suitable to reflect the bioassay results, as these could provide a different outcome [38]. In our study, at 5 min exposure to Temprid SC, both Tanjong Tokong and Green Lane strains showed a similar resistance level, demonstrating 50% mortality at 4 days post-treatment. However, we found that Green Lane strain was approximately four times more resistant than the Tanjong Tokong strain when RR_50_ values were compared. Likewise, Leong et al. [11] demonstrated that the *C. hemipterus* Kuala Lumpur strain was the least resistant towards Tandem when RR_50_ were compared. However, the Kuala Lumpur strain exhibited the lowest mortality in treatment with Tandem when compared to other tested populations. Deviation in the results between knockdown and mortality rate for the same resistant population may be due to the contributing resistance mechanisms in the insect population itself [38]. For instance, a resistant population that exhibited delayed or lower knockdown rate compared to mortality rate after treatment with a pyrethroid may have *kdr* as its major contributing resistance mechanism [38]. Besides, it is important to note that mortality data also should be recorded rather than just knockdown data as bed bugs can recover and feed, especially in trials with pyrethroid only based products [40]. Therefore, it is recommended to report both knockdown and mortality data to provide better interpretation of efficacy and resistance.

This study demonstrated that exposure time and mortality assessment intervals could influence time response bioassay results with bed bugs when tested at a single label rate. In our study, bed bugs were found to show differential responses with exposure time and mortality assessment intervals, resulting in different outcomes in RRs and mortalities. However, this may not apply to concentration-response bioassays that use a series of concentrations that result in mortalities between 0% and 100%. In a concentration-response bioassay, the time point for mortality assessment could affect lethal concentration (LC) and lethal dosage (LD) values. For example, a bioassay that records 24 h mortality is likely to show higher LC or LD values than that that registers mortality at 48 h. Nevertheless, the relative changes that occur in LC and LD values tested at different assessment intervals are likely similar for both susceptible and resistant strains. Hence, the assessment interval may unlikely affect the RR values of concentration-response bioassays significantly. More studies on this aspect are warranted.

Due to the lack of a known susceptible strain of *C. hemipterus*, we had to resort to the use of a susceptible strain of *C. lectularius* for comparison. It is assumed that both species do not have any inherent insecticide susceptibility differences, although we are unable to confirm this, and this may never be confirmed unless a susceptible *C. hemipterus* strain is established. In the past, other studies also had used a similar approach to evaluate insecticide resistance in *C. hemipterus* [5,11,41].

We propose that all insecticide product evaluations on bed bugs should involve at least one susceptible and two insecticide-resistant strains for comparisons. Knockdown responses and mortalities data should be recorded up to 96 h for fast-acting insecticides and up to 14 days for slow-acting insecticides. Due to the cryptic nature of bed bugs, the insects may not spend much time on a treated surface in the field. Therefore, a shorter exposure time (probably <1 h) is recommended to better reflect the possible field exposure conditions on bed bugs.

## 5. Conclusions

Exposure times and mortality assessment intervals have significant influences on bioassays results of tests carried out using single label rates of insecticide products. Knockdown responses and mortalities of the resistant strains were substantially higher with longer exposure times on treated surfaces and longer time intervals for mortality assessment. Further investigations using technical grade active ingredients and more insecticide-resistant bed bug populations may be necessary to further substantiate the present findings.

## Figures and Tables

**Table 1:** Bed bug strains evaluated in this study.

Species	Strain	Location Collected	Year Collected
*C. lectularius*	Monheim	Lab colony, Monheim, Germany	≈Late 1960s
*C. hemipterus*	Madam Mo	Collected from apartment	2016
	Tanjong Tokong	Collected from foreign worker dormitory	2015
	Green Lane	Collected from nursing home	2015

**Table 2:** Insecticide products used in this study.

Insecticide Class	Trade Name	Active Ingredient (%)	Application Rate (mg/m^2^)
Pyrethroid-neonicotinoid mixture	Tandem	Thiamethoxam (11.6%), lambda-cyhalothrin (3.5%)	183.96
	Temprid SC	Imidacloprid (21%), betacyfluthrin (10.5%)	106.13
Pyrethroid	Pesguard FG161	D-tetramethrin (4.4%), cyphenothrin (13.2%)	110
	Sumithrin	D-phenothrin (10%)	100
Organophosphate	Sumithion	Fenitrothion (20%)	250
Pyrrole	Phantom	Chlorfenapyr (24%)	300

**Table 3:** Classification of resistance level based on resistance ratio (RR_50_) [11,35].

Resistance Ratio (RR_50_)	Classification of Resistance Level
≤1	No resistance
>1 to ≤5	Low resistance
>5 to ≤10	Moderate resistance
>10 to ≤50	High resistance
>50	Very high resistance

**Table 4:** The KT_50_ and KT_95_ values of a susceptible *Cimex lectularius* strain and three *Cimex hemipterus* field strains tested using five insecticidal products (Tandem, Temprid SC, Pesguard FG161, Sumithrin, and Sumithion) applied at label rates.

Product	Strain	Exposure Time	KT_50_ (95% FL) (min)	KT_95_ (95% FL) (min)	Slope ± SE	χ^2^ (df)	RR_50_
Tandem	Monheim	5 min	16.1 (15.3–17.0)	25.0 (22.9–28.4)	8.7 ± 1.0	2.5 (6)	-
		10 min	16.3 (15.3–17.4)	30.3 (27.2–35.0)	6.1 ± 0.5	3.0 (9)	-
		30 min	15.1 (14.2–16.2)	28.0 (24.5–34.0)	6.1 ± 0.6	4.0 (9)	-
		1 h	16.3 (15.1–17.6)	31.9 (27.8–39.2)	5.6 ± 0.6	2.4 (6)	-
		4 h	15.4 (14.3–16.6)	29.8 (26.1–35.9)	5.8 ± 0.6	1.8 (7)	-
		Continuous	14.6 (13.7–15.8)	26.7 (23.3–32.4)	6.3 ± 0.7	1.3 (7)	-
	Madam Mo	5 min	15.0 (14.3–15.8)	24.6 (22.5–27.9)	7.6 ± 0.7	4.7 (9)	0.9
		10 min	16.2 (15.3–17.1)	29.5 (26.4–34.8)	6.3 ± 0.7	7.7 (9)	1.0
		30 min	15.8 (15.0–16.7)	29.3 (26.4–33.8)	6.1 ± 0.5	4.1 (13)	1.0
		1 h	15.6 (14.8–16.5)	28.9 (25.9–33.7)	6.2 ± 0.6	3.7 (10)	1.0
		4 h	15.7 (14.8–16.6)	29.6 (26.3–34.9)	5.9 ± 0.6	2.9 (10)	1.0
		Continuous	15.8 (14.9–16.8)	30.9 (27.4–36.7)	5.7 ± 0.6	5.6 (10)	1.1
	Tanjong Tokong	5 min	33.6 (31.1–36.3)	104.3 (88.1–130.5)	3.3 ± 0.3	1.4 (19)	2.1
		10 min	29.2 (26.8–32.0)	85.1 (70.4–110.8)	3.5 ± 0.3	1.9 (13)	1.8
		30 min	28.2 (26.0–30.7)	82.0 (68.9–104.2)	3.6 ± 0.3	5.2 (14)	1.9
		1 h	27.9 (25.7–30.4)	80.6 (67.6–102.4)	3.6 ± 0.3	2.3 (14)	1.7
		4 h	31.6 (28.8–34.6)	91.0 (76.2–115.8)	3.6 ± 0.3	2.5 (12)	2.1
		Continuous	29.2 (26.6–32.0)	97.8 (81.2–125.5)	3.1 ± 0.3	3.3 (15)	2
	Green Lane	5 min	57.6 (53.3–61.5)	124.0 (109.2–148.7)	4.9 ± 0.5	4.3 (11)	3.6
		10 min	49.7 (45.9–53.4)	115.6 (100.7–140.6)	4.5 ± 0.4	3.6 (11)	3.0
		30 min	43.3 (40.1–46.6)	101.1 (87.8–122.9)	4.4 ± 0.4	3.0 (11)	2.9
		1 h	39.5 (37.4–41.6)	80.6 (73.1–91.5)	5.3 ± 0.4	2.5 (17)	2.4
		4 h	39.2 (37.0–41.4)	83.7 (74.8–97.5)	5.0 ± 0.4	4.6 (16)	2.5
		Continuous	39.2 (37.3–41.2)	86.7 (77.7–100.0)	4.8 ± 0.4	4.5 (21)	2.7
Temprid SC	Monheim	5 min	15.9 (15.1–16.6)	26.7 (24.5–29.9)	7.3 ± 0.6	2.9 (11)	-
		10 min	17.0 (16.1–17.9)	28.3 (25.5–33.3)	7.4 ± 1.0	3.6 (7)	-
		30 min	16.2 (15.2–17.3)	29.4 (26.2–34.5)	6.4 ± 0.6	2.4 (7)	-
		1 h	16.4 (15.3–17.4)	32.3 (28.5–38.7)	5.6 ± 0.6	3.7 (9)	-
		4 h	17.3 (16.3–18.3)	31.2 (28.3–35.6)	6.4 ± 0.6	2.1 (10)	-
		Continuous	16.7 (15.6–17.9)	31.7 (28.2–37.2)	5.9 ± 0.6	4.4 (8)	-
	Madam Mo	5 min	16.3 (15.5–17.0)	26.3 (24.3–29.2)	7.8 ± 0.7	1.5 (11)	1.0
		10 min	17.0 (16.1–17.9)	28.6 (25.7–34.0)	7.3 ± 0.9	6.1 (7)	1.0
		30 min	16.5 (15.7–17.4)	27.8 (25.2–32.1)	7.3 ± 0.8	3.0 (8)	1.0
		1 h	15.8 (14.9–16.6)	27.8 (25.2–31.7)	6.7 ± 0.6	1.5 (10)	1.0
		4 h	16.1 (15.2–16.9)	28.0 (25.5–31.9)	6.8 ± 0.6	1.7 (10)	0.9
		Continuous	16.1 (15.3–16.9)	26.5 (24.1–30.6)	7.5 ± 0.8	1.8 (9)	1.0
	Tanjong Tokong	5 min	168.2 (133.3–218.3)	2890.9 (1572.8–7402.3)	1.3 ± 0.2	1.4 (11)	10.6
		10 min	201.3 (160.8–259.0)	4770.9 (2633.3–11159.0)	1.2 ± 0.1	3.5 (16)	11.8
		30 min	183.5 (139.9–255.6)	4295.4 (2009.2–14846.2)	1.2 ± 0.1	0.8 (10)	11.3
		1 h	52.3 (47.2–57.8)	174.0 (144.2–222.6)	3.1 ± 0.2	6.6 (13)	3.2
		4 h	60.1 (51.3–68.6)	225.2 (183.8–299.7)	2.9 ± 0.3	7.5 (9)	3.5
		Continuous	68.4 (61.0–75.7)	217.1 (118.6–278.2)	3.3 ± 0.3	4.8 (11)	4.1
	Green Lane	5 min	691.2 (520.5–1056.8)	7437.7 (3539.9–28224.0)	1.6 ± 0.2	1.0 (7)	43.7
		10 min	638.3 (503.0–915.0)	5080.6 (2644.2–17058.0)	1.8 ± 0.3	2.5 (7)	37.5
		30 min	415.2 (339.1–519.7)	3353.0 (2113.0–6820.6)	1.8 ± 0.2	0.9 (10)	25.2
		1 h	279.2 (232.0–342.5)	2273.3 (1482.2–4265.5)	1.8 ± 0.2	0.9 (10)	17.7
		4 h	60.4 (56.5–64.4)	124.0 (110.3–145.3)	5.2 ± 0.5	2.2 (11)	3.8
		Continuous	56.4 (52.4–60.4)	130.9 (115.4–155.1)	4.5 ± 0.4	2.5 (13)	3.5
Pesguard FG161	Monheim	5 min	8.0 (7.5–8.5)	14.5 (13.2–16.6)	6.3 ± 0.6	3.4 (9)	-
		10 min	7.4 (6.8–7.9)	14.2 (12.6–17.2)	5.8 ± 0.7	3.1 (7)	-
		30 min	7.6 (7.0–8.1)	14.2 (12.6–16.9)	6.0 ± 0.7	2.4 (7)	-
		1 h	7.7 (7.1–8.2)	16.4 (14.4–20.0)	5.0 ± 0.5	3.5 (9)	-
		4 h	8.0 (7.5–8.5)	14.2 (12.9–16.3)	6.7 ± 0.7	0.7 (8)	-
		Continuous	8.1 (7.5–8.6)	15.1 (13.5–17.7)	6.1 ± 0.7	0.8 (8)	-
	Madam Mo	5 min	13.0 (11.7–14.1)	23.6 (20.8–28.9)	6.3 ± 0.8	1.8 (4)	1.6
		10 min	10.0 (8.9–11.1)	21.1 (17.5–29.3)	5.0 ± 0.8	1.2 (3)	1.4
		30 min	9.1 (8.4–9.8)	18.6 (16.0–23.4)	5.3 ± 0.6	3.5 (8)	1.2
		1 h	10.1 (9.5–10.8)	18.3 (16.2–22.1)	6.4 ± 0.7	2.5 (7)	1.3
		4 h	9.1 (8.5–9.8)	17.3 (15.2–21.0)	5.9 ± 0.6	3.1 (8)	1.1
		Continuous	9.4 (8.8–9.9)	17.3 (15.5–20.2)	6.2 ± 0.6	2.0 (9)	1.2
	Tanjong Tokong	5 min	>5760.0	>5760.0	ND	ND	>720.0
		10 min	>5760.0	>5760.0	ND	ND	>778.4
		30 min	>5760.0	>5760.0	ND	ND	>757.9
		1 h	>5760.0	>5760.0	ND	ND	>748.1
		4 h	>5760.0	>5760.0	ND	ND	>720.0
		Continuous	778.1 (336.6–2594.6)	>5760.00	0.5 ± 0.1	2.8 (5)	96.1
	Green Lane	5 min	>5760.0	>5760.0	ND	ND	>720.0
		10 min	>5760.0	>5760.0	ND	ND	>778.4
		30 min	>5760.0	>5760.0	ND	ND	>757.9
		1 h	>5760.0	>5760.0	ND	ND	>748.1
		4 h	>5760.0	>5760.0	ND	ND	>720.0
		Continuous	>5760.0	>5760.0	ND	ND	>711.1
Sumithrin	Monheim	5 min	11.0 (10.3–11.6)	19.2 (17.2–22.3)	6.8 ± 0.7	2.1 (8)	-
		10 min	11.1 (10.5–11.7)	19.8 (18.1–22.4)	6.5 ± 0.6	1.5 (11)	-
		30 min	10.7 (10.1–11.3)	19.3 (17.5–22.2)	6.4 ± 0.6	2.0 (10)	-
		1 h	10.3 (9.7–10.9)	18.6 (16.7–21.8)	6.4 ± 0.7	3.0 (9)	-
		4 h	10.7 (10.1–11.3)	19.1 (17.1–22.5)	6.5 ± 0.7	2.1 (8)	-
		Continuous	10.8 (10.2–11.4)	17.9 (16.4–20.3)	7.5 ± 0.8	1.6 (8)	-
	Madam Mo	5 min	30.6 (28.0–33.3)	103.9 (86.3–134.1)	3.1 ± 0.3	0.4 (17)	2.8
		10 min	28.0 (25.5–30.5)	94.8 (76.8–129.4)	3.1 ± 0.3	1.5 (15)	2.5
		30 min	28.4 (26.3–30.5)	77.4 (66.3–94.8)	3.8 ± 0.3	1.4 (16)	2.7
		1 h	27.3 (25.6–29.0)	62.1 (55.3–72.4)	4.6 ± 0.4	3.8 (16)	2.7
		4 h	28.6 (26.9–30.4)	66.8 (59.3–78.0)	4.5 ± 0.3	3.3 (17)	2.7
		Continuous	25.8 (23.3–28.3)	64.5 (55.7–79.3)	4.1 ± 0.4	1.5 (9)	2.4
	Tanjong Tokong	5 min	>5760.0	>5760.0	ND	ND	>523.6
		10 min	>5760.0	>5760.0	ND	ND	>518.9
		30 min	>5760.0	>5760.0	ND	ND	>538.3
		1 h	>5760.0	>5760.0	ND	ND	>559.2
		4 h	>5760.0	>5760.0	ND	ND	>538.3
		Continuous	5367.7 (3642.1–10435.0)	>5760	1.1 ± 0.2	3.9 (7)	497.0
	Green Lane	5 min	>5760.0	>5760.0	ND	ND	>523.6
		10 min	>5760.0	>5760.0	ND	ND	>518.9
		30 min	>5760.0	>5760.0	ND	ND	>538.3
		1 h	>5760.0	>5760.0	ND	ND	>559.2
		4 h	>5760.0	>5760.0	ND	ND	>538.3
		Continuous	>5760.0	>5760.0	ND	ND	>533.3
Sumithion	Monheim	5 min	157.2 (145.7–167.3)	294.4 (264.9–344.4)	6.0 ± 0.7	3.7 (9)	-
		10 min	133.3 (112.3–147.9)	306.8 (264.3–402.1)	4.5 ± 0.7	2.4 (8)	-
		30 min	137.1 (129.2–143.8)	243.8 (224.3–274.7)	6.6 ± 0.7	2.3 (14)	-
		1 h	128.5 (119.9–136.7)	207.1 (189.5–234.2)	6.2 ± 0.6	1.3 (8)	-
		4 h	113.5 (106.0–121.1)	221.5 (197.6–259.7)	5.7 ± 0.5	2.3 (9)	-
		Continuous	116.4 (109.5–123.5)	211.3 (189.0–248.9)	6.4 ± 0.7	1.7 (8)	-
	Madam Mo	5 min	>5760.0	>5760.0	ND	ND	>36.6
		10 min	>5760.0	>5760.0	ND	ND	>43.2
		30 min	>5760.0	>5760.0	ND	ND	>42.0
		1 h	>5760.0	>5760.0	ND	ND	>44.8
		4 h	>5760.0	>5760.0	ND	ND	>50.7
		Continuous	698.8 (653.6–743.8)	1207.3 (1087.2–1405.9)	5.8 ± 0.5	1.9 (6)	6.0
	Tanjong Tokong	5 min	>5760.0	>5760.0	ND	ND	>36.6
		10 min	>5760.0	>5760.0	ND	ND	>43.2
		30 min	>5760.0	>5760.0	ND	ND	>42.0
		1 h	>5760.0	>5760.0	ND	ND	>44.8
		4 h	>5760.0	>5760.0	ND	ND	>50.7
		Continuous	921.7 (812.8–1035.3)	3713.7 (3045.2–4823.5)	2.7 ± 0.2	1.4 (13)	7.9
	Green Lane	5 min	>5760.0	>5760.0	ND	ND	>36.6
		10 min	>5760.0	>5760.0	ND	ND	>43.2
		30 min	>5760.0	>5760.0	ND	ND	>42.0
		1 h	>5760.0	>5760.0	ND	ND	>44.8
		4 h	>5760.0	>5760.0	ND	ND	>50.7
		Continuous	702.7 (638.1–767.3)	1450.2 (1250.0–1819.3)	5.2 ± 0.6	3.4 (5)	6.0

Knockdown responses of bed bugs were observed for 4 days. ND indicates no determined due to no/low knockdown rate and mortality.

**Table 5:** The KT_50_ and KT_95_ values of a susceptible *C. lectularius* strain and three *C. hemipterus* field strains tested using Phantom applied at label rate.

Strain	Exposure Time	KT_50_ (95% FL) (h)	KT_95_ (95% FL) (h)	Slope ± SE	χ^2^ (df)	RR_50_
Monheim	5 min	24.0 (23.9–24.2)	24.9 (24.8–25.2)	104.7 ± 12.9	1.0 (5)	-
	10 min	23.7 (23.6–23.9)	24.8 (24.6–25.2)	85.88 ± 12.1	3.8 (4)	-
	30 min	23.2 (23.0–23.4)	25.3 (24.9–25.9)	44.0 ± 4.6	4.2 (7)	-
	1 h	22.6 (22.3–22.9)	25.7 (25.0–26.7)	30.2 ± 3.7	2.4 (8)	-
	4 h	22.8 (22.4–23.1)	24.9 (24.3–25.9)	41.9 ± 4.3	9.4 (7)	-
	Continuous	21.4 (20.2–22.2)	25.6 (24.3–29.1)	21.1 ± 3.1	5.0 (4)	-
Madam Mo	5 min	36.1 (32.5–39.6)	84.9 (72.8–106.8)	4.4 ± 0.5	3.3 (7)	1.5
	10 min	30.4 (26.7–34.4)	91.8 (73.1–130.5)	3.4 ± 0.4	2.7 (6)	1.3
	30 min	32.0 (29.0–35.1)	77.6 (66.2–96.9)	4.3 ± 0.4	6.3 (8)	1.4
	1 h	31.6 (27.5–35.7)	79.2 (64.5–108.7)	4.1 ± 0.5	7.2 (7)	1.4
	4 h	25.8 (21.2–29.7)	95.8 (75.9–143.2)	2.9 ± 0.4	2.7 (8)	1.1
	Continuous	23.9 (19.7–27.7)	102.9 (75.9–175.6)	2.6 ± 0.4	1.9 (7)	1.1
Tanjong Tokong	5 min	32.6 (28.7–36.6)	108.7 (95.3–154.5)	3.1 ± 0.4	2.0 (8)	1.4
	10 min	38.4 (34.8–42.5)	102.6 (85.1–133.9)	3.8 ± 0.4	0.9 (8)	1.6
	30 min	22.4 (19.1–25.1)	60.4 (47.8–95.8)	3.8 ± 0.6	1.0 (5)	1.0
	1 h	20.8 (16.8–23.9)	67.6 (53.0–106.8)	3.2 ± 0.5	3.6 (6)	0.9
	4 h	21.6 (16.5–25.5)	95.8 (67.7–199.5)	2.5 ± 0.5	1.6 (6)	0.9
	Continuous	18.9 (16.1–21.3)	57.0 (45.4–84.2)	3.4 ± 0.5	2.5 (6)	0.9
Green Lane	5 min	>336.0	>336.0	ND	ND	>14.0
	10 min	>336.0	>336.0	ND	ND	>14.2
	30 min	332.0 (235.0–736.3)	>336.0	1.5 ± 0.4	2.0 (6)	14.3
	1 h	284.9 (202.3–769.1)	>336.0	1.2 ± 0.4	0.8 (6)	12.6
	4 h	48.4 (39.2–57.8)	378.9 (272.2–612.8)	1.8 ± 0.2	1.1 (11)	2.1
	Continuous	28.3 (22.4–33.9)	81.2 (58.8–173.4)	3.6 ± 0.5	6.4 (5)	1.3

Knockdown responses of bed bugs were observed for 14 days. ND indicates not determined due to no/low knockdown rate and mortality.

## Supplementary Materials

The following are available online at https://www.mdpi.com/2075-4450/11/9/640/s1, Table S1: Percentage mortality of a susceptible *C. lectularius* strain and three *C. hemipterus* strains exposed to Tandem, Temprid SC, Pesguard FG161, Sumithrin, and Sumithion at label rate. Table S2: Percentage mortality of a susceptible *C. lectularius* strain and three *C. hemipterus* strains exposed to Phantom at label rate.
