# Supplementary material for: Influences of Exposure Time and Mortality Assessment Interval on Bioassay Results of Insecticide-Resistant Tropical Bed Bugs (Hemiptera: Cimicidae)
Source: Insects. 2020 Sep 18;11(9):640. doi: 10.3390/insects11090640 (PMC7565302; doi:10.3390/insects11090640)
Supplement: Supplementary file 1 [file insects-11-00640-s001.pdf]

Supplementary Materials

Table S1. Percentage mortality of a susceptible *C. lectularius* strain and three *C. hemipterus* strains exposed to Tandem, Temprid SC, Pesguard FG161, Sumithrin, and Sumithion at label rate.

Product	Strain	Exposure time	Percentage mortality observed at different time interval post treatment (mean ± SE)			
			1-day	2-day	3-day	4-day
Tandem	Monheim	5 min	100a	100a	100a	100a
		10 min	100a	100a	100a	100a
		30 min	100a	100a	100a	100a
		1 h	100a	100a	100a	100a
		4 h	100a	100a	100a	100a
		Continuous	100a	100a	100a	100a
	Madam Mo	5 min	93.3 ± 6.7a	100a	100a	100a
		10 min	100a	100a	100a	100a
		30 min	100a	100a	100a	100a
		1 h	100a	100a	100a	100a
		4 h	100a	100a	100a	100a
		Continuous	100a	100a	100a	100a
	Tanjong Tokong	5 min	83.3 ± 3.3a	100a	100a	100a
		10 min	93.3 ± 6.7a	100a	100a	100a
		30 min	100a	100a	100a	100a
		1 h	100a	100a	100a	100a
		4 h	100a	100a	100a	100a
		Continuous	100a	100a	100a	100a
	Green Lane	5 min	86.7 ± 3.3a	96.7 ± 3.3a	100a	100a
		10 min	93.3 ± 6.7a	100a	100a	100a
		30 min	100a	100a	100a	100a
		1 h	100a	100a	100a	100a
		4 h	100a	100a	100a	100a
		Continuous	100a	100a	100a	100a
	Monheim	5 min	100a	100a	100a	100a

Temprid SC		10 min	100a	100a	100a	100a
		30 min	100a	100a	100a	100a
		1 h	100a	100a	100a	100a
		4 h	100a	100a	100a	100a
		Continuous	100a	100a	100a	100a
	Madam Mo	5 min	100a	100a	100a	100a
		10 min	100a	100a	100a	100a
		30 min	100a	100a	100a	100a
		1 h	100a	100a	100a	100a
		4 h	100a	100a	100a	100a
		Continuous	100a	100a	100a	100a
	Tanjong Tokong	5 min*	33.3 ± 3.3b	36.7 ± 3.3ab	46.7 ± 3.3ab	50.0a
		10 min*	53.3 ± 8.8b	56.7 ± 6.7ab	83.3 ± 8.8a	83.3 ± 8.8a
		30 min*	46.7 ± 3.3b	56.7 ± 8.8ab	76.7 ± 3.3a	76.7 ± 3.3a
		1 h	100a	100a	100a	100a
		4 h	100a	100a	100a	100a
		Continuous	100a	100a	100a	100a
	Green Lane	5 min	40a	40a	43.3 ± 3.3a	50 ± 5.8a
		10 min	40 ± 5.8a	40 ± 5.8a	53.3 ± 3.3a	60 ± 5.8a
		30 min	63.3 ± 8.8a	63.3 ± 8.8a	63.3 ± 8.8a	76.7 ± 6.7a
		1 h	53.3 ± 6.7a	56.7 ± 8.8a	70a	73.3 ± 3.3a
		4 h	70a	76.7 ± 3.3a	76.7 ± 3.3a	76.7 ± 3.3a
		Continuous	100a	100a	100a	100a
Pesguard FG161	Monheim	5 min	100a	100a	100a	100a
		10 min	100a	100a	100a	100a
		30 min	100a	100a	100a	100a
		1 h	100a	100a	100a	100a
		4 h	100a	100a	100a	100a
		Continuous	100a	100a	100a	100a
	Madam Mo	5 min	86.7 ± 3.3a	100a	100a	100a
		10 min	100a	100a	100a	100a
		30 min	100a	100a	100a	100a

		1 h	100a	100a	100a	100a
		4 h	100a	100a	100a	100a
		Continuous	100a	100a	100a	100a
	Tanjong Tokong	5 min	0a	0a	0a	0a
		10 min	0a	0a	0a	0a
		30 min	6.7 ± 3.3a	6.7 ± 3.3a	6.7 ± 3.3a	6.7 ± 3.3a
		1 h	23.3 ± 3.3a	23.3 ± 3.3a	23.3 ± 3.3a	23.3 ± 3.3a
		4 h	30 ± 11.5a	30 ± 11.5a	30 ± 11.5a	30 ± 11.5a
		Continuous*	33.3 ± 6.7b	33.3 ± 6.7b	63.3 ± 6.7ab	66.7 ± 8.8a
	Green Lane	5 min	0a	0a	0a	0a
		10 min	0a	0a	0a	0a
		30 min	0a	0a	0a	0a
		1 h	0a	0a	0a	0a
		4 h	0a	0a	0a	6.7 ± 3.3a
		Continuous*	0b	3.3 ± 3.3b	13.3 ± 3.3ab	30 ± 5.8a
Sumithrin	Monheim	5 min	100a	100a	100a	100a
		10 min	100a	100a	100a	100a
		30 min	100a	100a	100a	100a
		1 h	100a	100a	100a	100a
		4 h	100a	100a	100a	100a
		Continuous	100a	100a	100a	100a
	Madam Mo	5 min*	60 ± 5.8b	83.3 ± 6.7ab	100a	100a
		10 min*	56.7 ± 8.8b	90ab	100a	100a
		30 min	80a	100a	100a	100a
		1 h	83.3 ± 3.3a	100a	100a	100a
		4 h	86.7 ± 3.3a	100a	100a	100a
		Continuous	86.7 ± 3.3a	100a	100a	100a
	Tanjong Tokong	5 min	0a	0a	0a	0a
		10 min	0a	0a	0a	0a
		30 min	0a	0a	0a	0a
		1 h	3.3 ± 3.3a	3.3 ± 3.3a	3.3 ± 3.3a	3.3 ± 3.3a
		4 h	6.7 ± 3.3a	6.7 ± 3.3a	6.7 ± 3.3a	10 ± 5.8a

		Continuous*	20 ± 5.8b	36.7 ± 8.8 ab	53.3 ± 8.8a	56.7 ± 8.8a
	Green Lane	5 min	0a	0a	0a	0a
		10 min	0a	0a	0a	0a
		30 min	0a	0a	0a	0a
		1 h	0a	0a	0a	0a
		4 h	0a	0a	6.7 ± 3.3a	6.7 ± 3.3a
		Continuous*	6.7 ± 3.3b	16.7 ± 8.8ab	23.3 ± 3.3ab	30a
Sumithion	Monheim	5 min	100a	100a	100a	100a
		10 min	100a	100a	100a	100a
		30 min	100a	100a	100a	100a
		1 h	100a	100a	100a	100a
		4 h	100a	100a	100a	100a
		Continuous	100a	100a	100a	100a
	Madam Mo	5 min	0a	0a	6.7 ± 3.3a	10a
		10 min	0a	6.7 ± 3.3a	10 ± 5.8a	10 ± 5.8a
		30 min*	0b	10ab	16.7 ± 3.3a	20a
		1 h*	10 ± 5.8b	10 ± 5.8b	20 ± 5.8ab	23.3 ± 3.3a
		4 h	20 ± 5.8a	33.3 ± 8.8a	40 ± 5.8a	40 ± 5.8a
		Continuous	100a	100a	100a	100a
	Tanjong Tokong	5 min	0a	0a	0a	0a
		10 min	0a	10a	10a	10a
		30 min*	0b	10ab	10ab	13.3 ± 3.3a
		1 h	10a	10a	10a	13.3 ± 3.3a
		4 h	16.7 ± 6.7a	16.7 ± 6.7a	20.0 ± 5.8a	23.3 ± 8.8a
		Continuous*	70b	90ab	100a	100a
	Green Lane	5 min	0a	0a	0a	0a
		10 min	0a	0a	0a	6.7 ± 6.7a
		30 min	0a	3.3 ± 3.3a	6.7 ± 3.3a	10 ± 5.8a
		1 h	10a	10a	10a	10a
		4 h	6.7 ± 3.3a	10 ± 5.8a	10 ± 5.8a	16.7 ± 3.3a
		Continuous	100a	100a	100a	100a

*Percentage mortality across day 1 to day 4 is significantly different ($p < 0.05$; Friedman Test). Mean followed by different letters within the same row are significantly different ($p < 0.05$; Dunn's Test).

Table S2. Percentage mortality of a susceptible *C. lectularius* susceptible strain and three *C. hemipterus* strains exposed to Phantom at label rate.

Strain	Exposure time	Percentage mortality observed at different time point post treatment (mean ± SE)													
		**1-day**	**2-day**	**3-day**	**4-day**	**5-day**	**6-day**	**7-day**	**8-day**	**9-day**	**10-day**	**11-day**	**12-day**	**13-day**	**14-day**
Monheim	5 min*	46.7 ± 12.0b	100a	100a	100a	100a	100a	100a	100a	100a	100a	100a	100a	100a	100a
	10 min*	50 ± 11.5b	100a	100a	100a	100a	100a	100a	100a	100a	100a	100a	100a	100a	100a
	30 min	83.3 ± 8.8a	100a	100a	100a	100a	100a	100a	100a	100a	100a	100a	100a	100a	100a
	1 h	80 ± 5.8a	100a	100a	100a	100a	100a	100a	100a	100a	100a	100a	100a	100a	100a
	4 h	86.7 ± 6.7a	100a	100a	100a	100a	100a	100a	100a	100a	100a	100a	100a	100a	100a
	Continuous	93.3 ± 6.7a	100a	100a	100a	100a	100a	100a	100a	100a	100a	100a	100a	100a	100a
Madam Mo	5 min*	30 ± 5.8b	70 ± 5.8b	86.7 ± 6.7ab	100a	100a	100a	100a	100a	100a	100a	100a	100a	100a	100a
	10 min*	50 ± 17.3b	73.3 ± 6.7b	86.7 ± 8.8ab	96.7 ± 3.3ab	96.7 ± 3.3ab	100a	100a	100a	100a	100a	100a	100a	100a	100a
	30 min*	30 ± 11.6b	73.3 ± 6.7b	90 ± 5.8ab	90 ± 5.8ab	96.7 ± 3.3ab	100a	100a	100a	100a	100a	100a	100a	100a	100a
	1 h*	23.3 ± 14.5b	80b	90 ± 5.8ab	96.7 ± 3.3ab	100a	100a	100a	100a	100a	100a	100a	100a	100a	100a
	4 h*	46.7 ± 3.3b	70 ± 10.0b	90 ± 5.8ab	96.7 ± 3.3ab	100a	100a	100a	100a	100a	100a	100a	100a	100a	100a
	Continuous*	50 ± 10.0b	70 ± 10.0b	90ab	96.7 ± 3.3ab	100a	100a	100a	100a	100a	100a	100a	100a	100a	100a
Tanjong Tokong	5 min*	36.7 ± 6.7b	66.7 ± 12.0b	90ab	93.3 ± 3.3ab	100a	100a	100a	100a	100a	100a	100a	100a	100a	100a
	10 min*	23.3 ± 8.8b	63.3 ± 3.3b	86.7 ± 3.3ab	93.3 ± 6.7ab	100a	100a	100a	100a	100a	100a	100a	100a	100a	100a
	30 min*	53.3 ± 8.8b	90 ± 10.0ab	100a	100a	100a	100a	100a	100a	100a	100a	100a	100a	100a	100a
	1 h*	56.7 ± 12.0b	80ab	100a	100a	100a	100a	100a	100a	100a	100a	100a	100a	100a	100a
	4 h*	60 ± 11.6b	76.7 ± 3.3b	100a	100a	100a	100a	100a	100a	100a	100a	100a	100a	100a	100a
	Continuous*	56.7 ± 13.3b	93.3 ± 3.3ab	100a	100a	100a	100a	100a	100a	100a	100a	100a	100a	100a	100a

Green Lane	5 min*	0b	0b	10ab	20ab	20ab	23.3 ± 3.3ab	30a	30a	33.3 ± 3.3a	33.3 ± 3.3a	33.3 ± 3.3a	33.3 ± 3.3a	40a	40a
	10 min*	0c	6.7 ± 3.3bc	13.3 ± 3.3abc	23.3 ± 3.3abc	26.7 ± 3.3abc	26.7 ± 3.3abc	33.3 ± 8.8ab	33.3 ± 8.8ab	33.3 ± 8.8ab	33.3 ± 8.8ab	40 ± 10.0a	40 ± 10.0a	40 ± 10.0a	40 ± 10.0a
	30 min*	0c	6.7 ± 3.3bc	13.3 ± 8.8abc	23.3 ± 12.0abc	30 ± 5.8abc	33.3 ± 6.7abc	36.7 ± 3.3ab	36.7 ± 3.3ab	36.7 ± 3.3ab	36.7 ± 3.3ab	36.7 ± 3.3ab	40 ± 5.8a	46.7 ± 8.8a	46.7 ± 8.8a
	1 h*	0c	20bc	20bc	23.3 ± 3.3abc	30abc	33.3 ± 3.3abc	40 ± 10abc	40 ± 10abc	43.3 ± 8.8ab	46.7 ± 12.0a	46.7 ± 12.0a	53.3 ± 8.8a	53.3 ± 8.8a	53.3 ± 8.8a
	4 h*	26.7 ± 6.7d	56.7 ± 6.7cd	63.3 ± 8.8bcd	66.7 ± 6.7abcd	73.3 ± 6.7abcd	76.7 ± 3.3abcd	83.3 ± 3.3abcd	86.7 ± 3.3abc	90ab	90ab	93.3 ± 3.3a	93.3 ± 3.3a	100a	100a
	Continuous*	33.3 ± 8.8b	86.7 ± 3.3ab	96.7 ± 3.3ab	100a	100a	100a	100a	100a	100a	100a	100a	100a	100a	100a

* Percentage mortality across day 1 to day 14 is significantly different ($p < 0.05$; Friedman Test). Mean followed by different letters within the same row are significantly different ($p < 0.05$; Dunn's Test).
